# Magnetic properties of ferritin and akaganeite nanoparticles in aqueous suspension

**DOI:** 10.1007/s11051-013-1902-0

**Published:** 2013-08-14

**Authors:** Marceli Koralewski, Mikołaj Pochylski, Jacek Gierszewski

**Affiliations:** Optics Laboratory, Faculty of Physics, Adam Mickiewicz University, Umultowska 85, 61-614 Poznan, Poland

**Keywords:** Magnetic birefringence, Cotton–Mouton effect, Akaganeite, Ferritin, Magnetic susceptibility anisotropy, Langevin function

## Abstract

We have studied the magnetically induced optical birefringence Δ*n* of horse spleen ferritin (HSF) and aqueous suspensions of several different-sized iron oxyhydroxide nanoparticles coated with different polysaccharides mimicking ferritin. The structure and dimensions of the akaganeite mineral core were characterized by XRD and TEM, respectively. The stability of the suspensions in the measurement temperature range from 278 to 358 K was confirmed by UV–Vis absorption spectroscopy. The values of optical polarizability anisotropy Δ*α*, magnetic susceptibility anisotropy Δ*χ*, and permanent magnetic dipole moment *μ*_m_ of the akaganeite nanoparticles have been estimated on the basis of the temperature dependence of the Cotton–Mouton (C–M) constant. The magnetic birefringence of Fe-sucrose has been described tentatively by different types of Langevin function allowing another estimation of Δ*χ* and *μ*_m_. The obtained permanent magnetic dipole moment *μ*_m_ of the studied akaganeite nanoparticles proves small and comparable to that of HSF. The value of *μ*_m_ is found to increase with decreasing nanoparticle diameter. Observed in a range spanning more than five orders of magnitude, the linear relation between the C–M constant and the iron concentration provides a basis for possible analytical application of the C–M effect in biomedicine. The established relation between the C–M constant and the nanoparticle diameter confirms that the dominant contribution to the measured magnetic birefringence comes from the magnetic susceptibility anisotropy Δ*χ*. A comparison of the C–M constants of the studied akaganeite nanoparticles with the data obtained for HSF provides evidence that the ferritin core behaves as a non-Euclidian solid.

## Introduction

Essential for life processes, iron plays a very important role in living organisms of all species (Lindley 1996). It is stored by animals and plants, as well as bacteria, inside the protein ferritin (Harrison and Arosio 1996). Malfunction of the iron-storing mechanism of ferritin leads to serious, mostly blood diseases, the treatment of which requires the intravenous application of an iron-containing agent. Different polysaccharide iron complexes (PICs) are pharmaceutically important substitutes for ferritin, used as hematinics for the treatment of anemia (London 2004; Funk et al. 2001). Also, PICs are often used as model systems in physicochemical studies of ferritin. Although the mineral cores of PICs and ferritin have different unit cells, both have Fe^3+^ ions octahedrally coordinated by oxygen. Aside from purely pharmaceutical applications, the increasing interest in iron oxyhydroxide particles of desired size and chemical properties is also stimulated by their potential technological applications, especially as a low-cost adsorbing agent (Ibrahim et al. 1994; Kolbe et al. 2011; Xu et al. 2012).

A PIC system is an aqueous colloidal suspension composed of an iron oxyhydroxide-based core of a diameter of a few nanometers surrounded by a polysaccharide shell a few nanometer thick (London 2004; Funk et al. 2001). Besides the well-known iron dextran (London 2004; Lawrence 1998), commonly used as a drug and in research (Kilcone and Gorisek 1998), the commercially available PICs include, among others, Venofer^®^, a complex of polynuclear iron(III) oxyhydroxide in sucrose (Venofer^®^ 2011), Dexferrum^®^, a complex of iron oxyhydroxide stabilized by dextran (Dexferrum^®^ 2010), or Ferrum Lek, iron oxyhydroxide stabilized by polymaltose (Ferrum LEK^®^ 2010). Although the clinical applications of PICs are well documented in the literature (see, e.g., Venofer^®^ 2009), only a few studies focus on the physicochemical properties of these compounds (Funk et al. 2001; Gutierrez et al. 2005; Kudasheva et al. 2004; Knight et al. 1999). Polysaccharide iron complexes have been characterized by X-ray diffraction (XRD), iron-57 Mossbauer spectroscopy, transmission electron microscopy (TEM), atomic force microscopy (AFM), and other techniques (Ibrahim et al. 1994). Some magnetic properties were found in magnetization and susceptibility measurements (Gutierrez et al. 2005). In spite of the considerable experimental effort many properties of PIC systems remain to be explored and specified.

There is some controversy about the mineral constitution of the iron core. The X-ray diffraction data show that the structure of the core is the most consistent with the akaganeite polymorph β-FeOOH (Funk et al. 2001; Kudasheva et al. 2004; Knight et al. 1999; Kilcoyne and Lawrence 1999). However, some authors claim that the core has rather a 2-line ferrihydrite structure (Gutierrez et al. 2005). Others suppose (Coe et al. 1995a) an intermediate structure between that of akaganeite and ferrihydrite, which can appear to closely resemble both, depending on the technique used for structure probing. Most of the studies of PICs performed so far used freeze-dried samples in low-temperature conditions (Coe et al. 1995a, b; Oshtrakh et al. 2006). Studies of the liquid state of PIC suspensions at physiological temperature (310 K) or above room temperature (RT) are rare and mostly obscure. We have recently published preliminary data on the magnetic properties of the liquid hematinic iron sucrose aqueous suspension (Koralewski et al. 2011a). We have shown that magnetooptical effects can be of much use for the discrimination of the valency of iron and the description of magnetic characteristics of ferrofluids (Koralewski et al. 2011b, 2012a, b). The magnetic characteristics of ferritin and PIC compounds are of key interest for non-invasive assessment of iron in human body, e.g. by magnetic resonance imaging (MRI) or susceptometry, in the frame of emerging techniques in medical practice (Schenck and Zimmerman 2004; Carneiro et al. 2005).

In this study we present the magnetically-induced linear optical birefringence and its temperature dependence in several commercial hematinics in the form of aqueous suspension of iron oxyhydroxide nanoparticles with different core sizes. We use these data for determining the magnetic dipolar properties of the nanoparticles. The results obtained are compared with those of similar measurements performed for horse spleen ferritin (HSF), and discussed in the light of earlier investigations of similar nanoscale systems. The discussion of the results is supported by additional XRD, TEM, VSM, refractive index, and UV–Vis absorption spectrum measurements.

## Experimental

The iron dextran (Sigma) and parenteral iron formulations used in this study were iron oxyhydroxide polysaccharide aqueous suspensions specified in Table 1. Horse spleen ferritin was used as a natural biogenic ferritin. Several different lots of holoferritin and apoferritin were purchased from Sigma and were used without further processing. Pure dextran (5 kDa) was obtained from Pharmacosmos (Denmark). Ficoll^®^-70, a synthetic polymer of sucrose, and a maltose solution were purchased from Fluka.

**Table 1:** Selected physicochemical properties of the studied iron oxyhydroxide polysaccharide aqueous suspensions, with error in parentheses

Property	Fe-sucrose	Fe-polymaltose	Fe-dextran	Fe-dextran (S)
Trade name	Venofer^®^	Ferrum Lek^®^	Dexferrum^®^	Iron dextran
Supplier	Lek	Lek	American Regent	Sigma-Aldrich
Core	Akaganeite	Akaganeite	Akaganeite	Akaganeite
Carrier	Water	Water	Water	Water
Shell	Polysucrose	Polymaltose	Dextran	Dextran
Fe-content (g/L)^a^	20 (1.4)	50 (3.5)	50 (3.5)	100 (7)
Density (10^3 ^kg/m^3^)^a^	1.1576	1.0863	1.1483	1.1789
Refractive index^b^	1.3341	1.3317	1.3321	1.3321
Core diameter (nm)				
XRD^c^	~3.8 (0.7)	6.5	5.6	5.6
TEM	~3 (0.5)	6	4.5	5 × 16

^a^Mother liquor/aqueous suspension at RT

^b^ *λ* = 632.8 nm, RT, *c* ^Fe^ = 1 g/L

^c^Estimated for (211) diffraction peak

The iron content in the mother liquors, i.e., as-received original iron polysaccharide liquid suspensions, and in HSF was measured by inductively coupled plasma (ICP) optical emission spectrometry (OES) with a VISTA-MPX (Varian) spectrometer. The iron content in the sample was as claimed by the supplier (see Table 1) within the experimental error (less than 7 %). For optical studies the initial suspension was further diluted with triple-distilled demineralized water.

Room temperature X-ray diffraction (XRD) measurements were performed using CuKα (*λ* = 0.154056 nm) radiation with a D8 Advance (Bruker) diffractometer. The sample, i.e., the mother liquor suspension, was held in a glass capillary during the experiment. Transmission electron microscopy (TEM) analysis was performed with a JEM-1200 EX II (JEOL) microscope operated at 80 kV. Magnetization measurements were carried out at room temperature with a home-made vibrating sample magnetometer (VSM) (Koralewski et al. 2012a).

The optical birefringence was measured by means of a home-built polarimeter described in detail by Koralewski et al. (2011a). The instrument allows precise measurements of the angle *θ* of rotation of the polarization plane for light passing through a sample in a magnetic field in the Voigt configuration. The sample was held in a jacketed cylindrical glass cell with a pathlength ranging from 2 to 100 mm, depending on the iron concentration in the sample. A Haake programmable refrigerant circulator (Thermo-Fisher) was used for temperature stabilization in the temperature range from 278 to 358 K with an accuracy better than 0.1 K. The light beam from a He–Ne laser (*λ* = 632.8 nm) was used.

The angle *θ* of rotation of incident polarized light is proportional to the birefringence Δ*n* of the sample by the well-known relation:

1 $$ \theta = \frac{\pi L}{\lambda }\Updelta n, $$

where *L* denotes the length of the cell and* λ* the wavelength of the light used. The accuracy of the angle estimation was better than 0.001º. This angular precision, together with utilization of sufficiently long sample cell, allows the magnetic birefringence Δ*n* to be measured with accuracy better than 10^−9^. The maximal feasible optical path, and so the precision of Δ*n*, was limited by the transparency of studied fluid (different for different concentrations of magnetic nanoparticles). For each sample examined many trials were performed to find optimal cell length minimizing the error in the Cotton–Mouton constant,* C* ^CM^. Depending on the range of the value of* C* ^CM^ and concentration of iron in fluid under examination we were able to determine the* C* ^CM^ with relative error from 0.05 to 10 %. In order to conduct an automated experiment the current supply, the gaussmeter, the refrigerant circulator, the analyzer rotary stage and the photomultiplier were all interfaced to a PC computer. A program written in LabVIEW^®^ was used for synchronizing the measurement sequence and for data collection.

The dispersion and the temperature dependence of the light refractive index *n* of diluted liquid iron polysaccharide suspensions were measured with a Pulfrich refractometer PR2 (Zeiss) equipped with a thermostatable prism allowing measurements of *n* in the temperature range from 278 to 318 K. The refractive index was measured with accuracy ±0.0001. The absorption spectra were measured with a computer-aided Specord M40 (Zeiss) spectrophotometer in the temperature range from 278 to 363 K using a jacketed quartz cell with a 10 mm pathlength. An MK 70 (Zeiss) thermostat with temperature control ±0.1 K was used in both cases. The estimated error for molar extinction coefficient is below 0.1 %. The density was measured using a DMA 38 (Anton Paar) density meter in the temperature range from 278 to 318 K with accuracy ±0.0001 g/cm^3^.

## Theoretical background

Since Majorana (1902) and Cotton and Mouton’s (1907) discovery magnetic field is known to induce molecular order via dipolar alignment. Hence, optical birefringence can occur in suspensions of anisotropic, anisometric, and diamagnetic particles. There are two types of origin of magnetic dipoles. The permanent magnetic dipole moment *μ*_m_ is inherent and characteristic of the molecular structure. A magnetic dipole moment can be also induced by a magnetic field. In the latter case the value of the magnetic dipole moment is related to the magnetic susceptibility and its anisotropy Δ*χ* in the material.

The analysis of the static optical birefringence of a dilute suspension of axially symmetrical particles follows the description proposed by (Peterlin and Stuart 1939), developed by O’Konski et al. (1952) and discussed by other authors (Wilson et al. 1998; Hassamony et al. 1989). In this approach, the optical birefringence induced by a continuous magnetic field of magnitude *B* is given by (in SI units):

2 $$ \Updelta n = \, \Updelta n_{\text{s}} F\left( {p,q} \right) \, = \frac{{\rho^{N} \Updelta \alpha }}{{2n\varepsilon_{\text{0}} }}\,F\left( {p,q} \right), $$

where Δ*n*_s_ is the saturation magnetic birefringence described by the anisotropy Δ*α* of volume optical polarizability, *ρ* ^N^ is the volume concentration (particle number density), *n* is the refractive index of the solution, *ε*_0_ denotes the permittivity of free space, and *F*(*p*, *q*) is a statistical particle orientation function. In the case of magnetic birefringence the parameters of *F*(*p*, *q*) correspond to the energy of interaction of the magnetic field with both the permanent and induced magnetic dipole moments. In general, in the low magnetic field strength limit, after O’Konski et al. (1952) the orientation function *F*(*p*, *q*) is given by:

3 $$ F = \, \left( {p^{ 2} + { 2}q} \right)/ 1 5, $$

where *p* = *μ*_m_ *B*/*kT*, *q* = Δ*χB* ^2^/(2*μ*_0_ *kT*), *μ*_0_ is the permeability of free space, *k* is the Boltzmann constant, and *T* stands for the absolute temperature.

Measurements in the range referred to as the low-field region, in which the birefringence changes as the square of the magnetic field *B*, allow direct calculation of the Cotton–Mouton constant *C* ^CM^ from the relation:

4 $$ \Updelta n = C^{CM} \lambda B^{ 2} . $$

Taking into account Eqs. (2), (3), and (4), the Cotton–Mouton constant can be written as:

5 $$ C^{{\rm CM}} = \frac{{\rho^{N} \Updelta \alpha }}{{30n\varepsilon_{\text{0}} \lambda }}\left\{ {\frac{\Updelta \chi }{{\mu_{\text{0}} kT}} + \left( {\frac{{\mu_{\text{m}} }}{kT}} \right)^{2} } \right\}. $$

As implied by Eq. (5), the values of Δ*χ* and *μ*_m_ can be determined from the temperature dependence of the Cotton–Mouton constant only if the value of Δ*n*_s_ or Δ*α* is known. Although Δ*α* can be measured in different experiments, its value can also be estimated from the saturation of the magnetic birefringence Δ*n*_s_ (observed at high magnetic fields) and the refractive index (see Eq. (2)). When the magnetic field is too low for full magnetic birefringence saturation to occur, an extrapolation of Δ*n* to the high-field limit allows to estimate Δ*n*_s_. In this case the saturation magnetic birefringence can be obtained from the Δ*n* versus 1/*B* ^2^ relation by graphical extrapolation.

The temperature dependence of the Cotton–Mouton constant can be linearized by plotting the product *GTC* ^CM^ versus 1/*T*. The parameter *G* relates to Δ*α* as $G = 30nk\varepsilon_{0} \lambda /\rho^{\rm N} \Updelta \alpha (T) = 1 5 k\lambda /\Updelta n_{\text{s}} \left( T \right)$ and can be assumed either constant (in which case a *TC* ^CM^ versus 1/*T* plot will suffice) or temperature-dependent (through Δ*n*_s_). The approaches based on these two assumptions, with Δ*n*_s_ temperature-independent or temperature-dependent, will be henceforth referred to as the first and second methods of linearization, respectively. In each case the values of Δ*χ* and *μ*_m_ of the particle can be calculated from the intercept and the slope of the linearized dependence. It needs to be stressed that both values strongly depend on the precision of estimation of Δ*α* (Δ*n*_s_).

In the case of complete (or nearly complete) ordering of the magnetic moments, when saturation of magnetic birefringence occurs, the phenomenon can be described by a second-order Langevin function *L*_2_ as the statistical orientation function figuring in Eq. (2) (Hassamony et al. 1989; Scholten 1975; Neitzel and Barner 1977). Taking into account only the contribution from the permanent magnetic moments to the magnetic orientation effects, the value of birefringence can be calculated from:

6 $$ \Updelta n = \Updelta n_{\text{s}} L_{2} (p) = \frac{{\rho^{N} \Updelta \alpha }}{{2n\varepsilon_{\text{0}} }}L_{2} (p), $$

where *L*_2_(*p*) = 1−3*L*_1_(*p*)/*p*, and *L*_1_(*p*) is the usual first-order Langevin function.

The value of *μ*_m_ can be obtained directly by fitting the Eq. (6) to the experimental static birefringence measured as a function of *B*. The value of magnetic field required to saturate the Langevin function also provides information on the average magnetic moment, since the field decreases with increasing magnetic moment.

The description of orientation phenomena can be generalized by allowing for contributions from both permanent and induced magnetic moments. As shown by Kielich (1970) in such case the *L*_2_ term depends on both *p* and *q*:

6a $$ \Updelta n = \, \Updelta n_{\text{s}} L_{ 2} \left( {p, \, \pm q} \right). $$

The sign of *q* refers to positive or negative anisotropy of magnetic susceptibility. Although the integral in the expression for *L*_2_(*p*, *q*) cannot be solved analytically (Kielich 1970; Scholten 1975), numerical procedures allow to obtain the values of *μ*_m_ and Δ*χ* by fitting the experimental Δ*n*(*B*) dependence to the Eq. (6a).

If the spherical shape of the particles can be assumed the value of permanent magnetic moment is related to the diameter *D* of each particle and its saturation magnetization *M*_s_ by the formula:

7 $$ \mu_{\text{m}} = M_{\text{s}} \pi D^{3} /6. $$

If *M*_S_ is known from independent measurements, Eq. (7) can be used for estimating the particle diameter *D* (and vice versa).

When the system is a polydisperse suspension Eq. (6) or (6a) need to be rewritten to take into account the particle size distribution:

8 $$ \Updelta n = \Updelta n_{\text{s}} \frac{{\int {L_{2} \,P(D)\,D^{3} \,dD} }}{{\int {P(D)\,D^{3} \,dD} }}, $$

where *L*_2_ can be *L*_2_(*p*) or *L*_2_(*p*, ± *q*), and P(*D*) is the most commonly used log-normal size distribution function defined as (Popplewell and Sakhnini 1995):

9 $$ P(D) = \frac{1}{{\sqrt {2\pi } sD}}\exp \left[ { - \frac{{\ln^{2} (D/D_{0} )}}{{2s^{2} }}} \right], $$

where *D*_0_ and *s* are parameters of the distribution function *P*(*D*), related to the average diameter 〈*D*〉 and the standard deviation* σ* by the formulas (Rasa 2000):

$$ \langle D \rangle \, = D_{0} {\exp}\left( {s^{2}/2} \right)\quad{\text{and}}\, \sigma = D_{0} {\exp}\left( {s^{2}/2}\right)\left( {{\exp}\;s^{2}-{1}} \right)^{1/2}. $$

## Results and discussion

### X-ray diffraction (XRD) and transmission electron microscopy (TEM) characterization

#### XRD

**Fig. 1:**
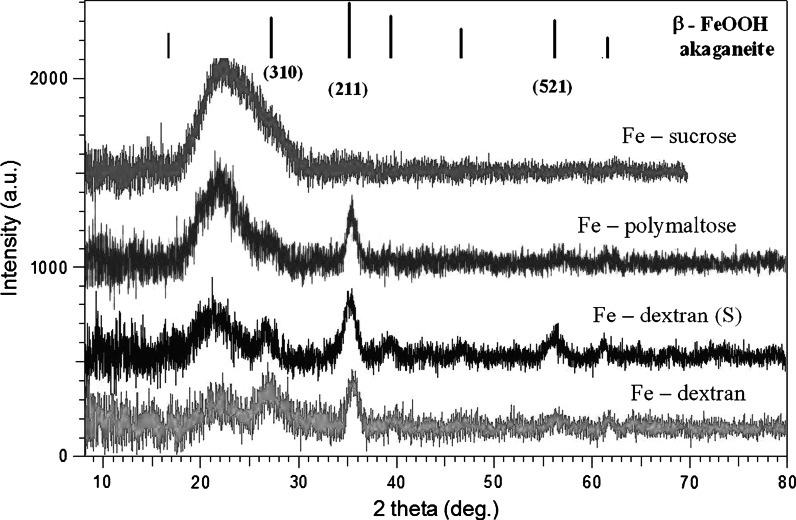
X-ray diffractograms of iron oxyhydroxide polysaccharide suspension in mother liquor solution in a quartz glass capillary. The *vertical bars* indicate the position of the main diffraction peak of akaganeite as specified in JCPDS # 34-1266

The X-ray diffraction patterns of the mother liquors (see Table 1) consist of several relatively broad peaks indicative of the β-FeOOH akaganeite (Cornell and Schwertmann 2003; Post et al. 2003) (Fig. 1). The diffraction pattern of dextran powder (not shown) consists of very broad peaks centered at 2*θ* ≈ 18° and much weaker peaks around 2*θ* ≈ 40°, which suggests not a crystalline, but rather a glassy state. This means that, in contrast to the iron oxide core material, dextran itself does not imply the crystalline phase. We have observed (diffractograms not shown) that in the presence of high excess of dextran coating in the solution the main peaks from the crystalline core material were overwhelmed by very broad peaks from dextran (a similar situation occurs in the case of Fe-sucrose, see Fig. 1). In such case the information provided by XRD patterns is less reliable than that acquired by other methods. However, when a solution with a higher iron content is studied (see Fig. 1, Fe-dextran (S)), the diffractogram is comparable in quality to those of classical freeze-dried samples (Kilcoyne and Lawrence 1999). The most pronounced diffraction peak, corresponding to the (211) plane (diffraction angle 2*θ* = 35.3°), was used with the Scherrer formula (Klug and Alexander 1974) for estimating the mean crystalline dimension of the studied compounds. The results are reported in Table 1. In both iron dextran (Fe-dextran(S) and Fe-dextran) samples the diffraction peak corresponding to the (310) plane (2*θ* = 26.7°) allowed to estimate the mean crystalline dimension related to this plane as well (about 3.8–3.9 nm). This means that nanoparticles of these samples are distorted from the ideal spherical shape, in agreement with reports of other authors (Knight et al. 1999; Kilcoyne and Lawrence 1999; Bashir et al. 2009). Note that the XRD patterns of the studied samples appear to have a weak peak at 2*θ* ≈ 61.15°, which would indicate the presence of Cl^−^ anions. This is consistent with the tunnel-containing akaganeite structure (Kudasheva et al. 2004; Kilcoyne and Lawrence 1999; Post et al. 2003).

#### TEM

**Fig. 2:**
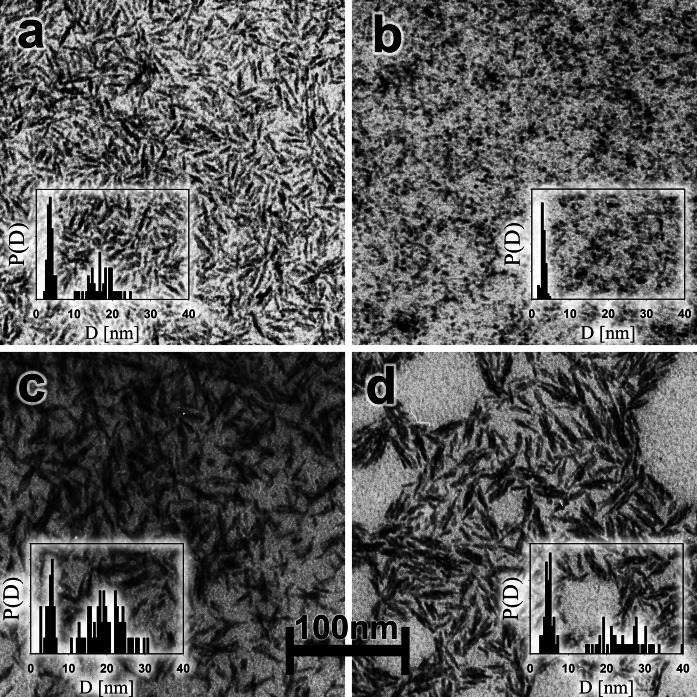
TEM micrographs of aqueous suspension of iron oxyhydroxide polysaccharides: **a** Fe-dextran (S), **b** Fe-sucrose, **c** Fe-dextran, **d** Fe-polymaltose. The *inset* shows the distribution of nanoparticles size

TEM micrographs of the studied suspensions are shown in Fig. 2. In this particular case, utilization of the dedicated software for automated particle size distribution analysis is not possible because the particles are crowded and overlaps. To gain information about their dimensions, the particles were manually identified and their size was determined from the number of pixels occupied in the image. At least 100 particles, in different areas of the image, has been subjected to the above analysis. This made possible to draw a histogram presenting particle size distribution. As shown in Fig. 2a, c, d in happened that the shape of particles was ellipsoidal. In these cases, sizes of major and minor ellipse semiaxis were determined, which are represented by two peaks in appropriate histograms (insets in Fig. 2). The average sizes of the nanoparticles of the studied compounds are specified in Table 1. Note that all the compounds (excluding Fe-sucrose) show also a broad spectrum of possible aggregation (dimmers, trimmers) with a rather low population of the aggregates. At present it cannot be excluded that the occurrence of “background” aggregates may result from the preparation of the sample for electron microscopy studies. Spindle-shaped particles with a size of (4 × 20 nm), approximately, have been already observed in Fe-dextran (S) by Lazaro et al. (2003). The data in Table 1 show a good agreement between the TEM and XRD results concerning the size of the mineral core. These results are also in very good agreement with those obtained for Fe-sucrose by Kudasheva et al. (2004), and for Fe-dextran (S) by other authors (Funk et al. 2001; Kilcoyne and Gorisek 1998).

### Optical characterization

#### Refractive index

**Fig. 3:**
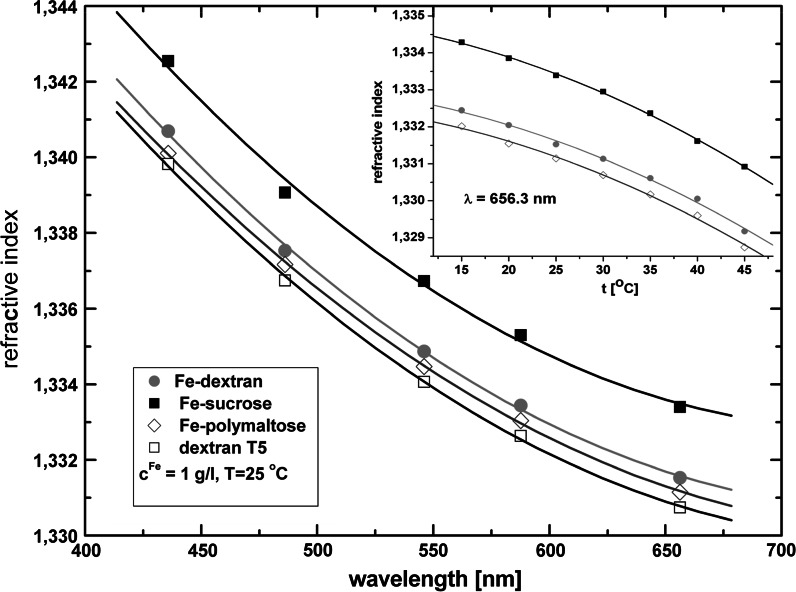
Refractive index dispersion for pure low molecular weight (5 kDa) dextran and iron oxyhydroxide polysaccharide aqueous suspensions studied at room temperature (*c* ^Fe^ = *c* ^P^ = 1 g/L). The *inset* shows the temperature dependence of the refractive index for *λ* = 656.3 nm. The wavelength and temperature dependences of *n* for Fe-dextran and Fe-dextran (S) are identical within the error (the size of the *error bar* is smaller than the dimension of the experimental point)

The refractive index measured for pure low molecular weight (5 kDa) dextran and iron oxyhydroxide polysaccharide suspensions for several wavelengths in the range from 405 to 656 nm at room temperature (RT) shows a usual dispersion. The results can be easily described by a Sellmeier-type Equation (Daimon and Masumura 2007) allowing the calculation of the refractive index at 632.8 nm, the wavelength used in the magnetic birefringence measurements (see Fig. 3; Table 1). In all the studied suspensions the index of refraction decreases monotonically with the temperature increasing to 313 K in a manner similar to that observed in pure water (see Fig. 3). Also the temperature dependence of the density (not shown) resembles that observed in pure water. The refractive index measurement data can be of use for estimating the polysaccharide concentration in the investigated samples. By assuming additional contributions of polysaccharide and akaganeite nanoparticles to the refractive index of the whole suspension we have established the concentration of sucrose and polymaltose equal to 340 g/L and lower than 50 g/L, respectively, in these two suspensions. In the calculation we used the measured refractive index increment coefficient d*n*/d*c* equal to 0.103 and 0.133 mL/g for pure sucrose (Ficoll^®^) and maltose solutions, respectively. In the case of Fe-dextran (S) the polymer concentration in the mother liquor was 100 g/L, as specified by the supplier. The equal values of *n* obtained for Fe-dextran and Fe-dextran (S) suggest similar polymer concentration in both solutions.

#### UV–Vis absorption

**Fig. 4:**
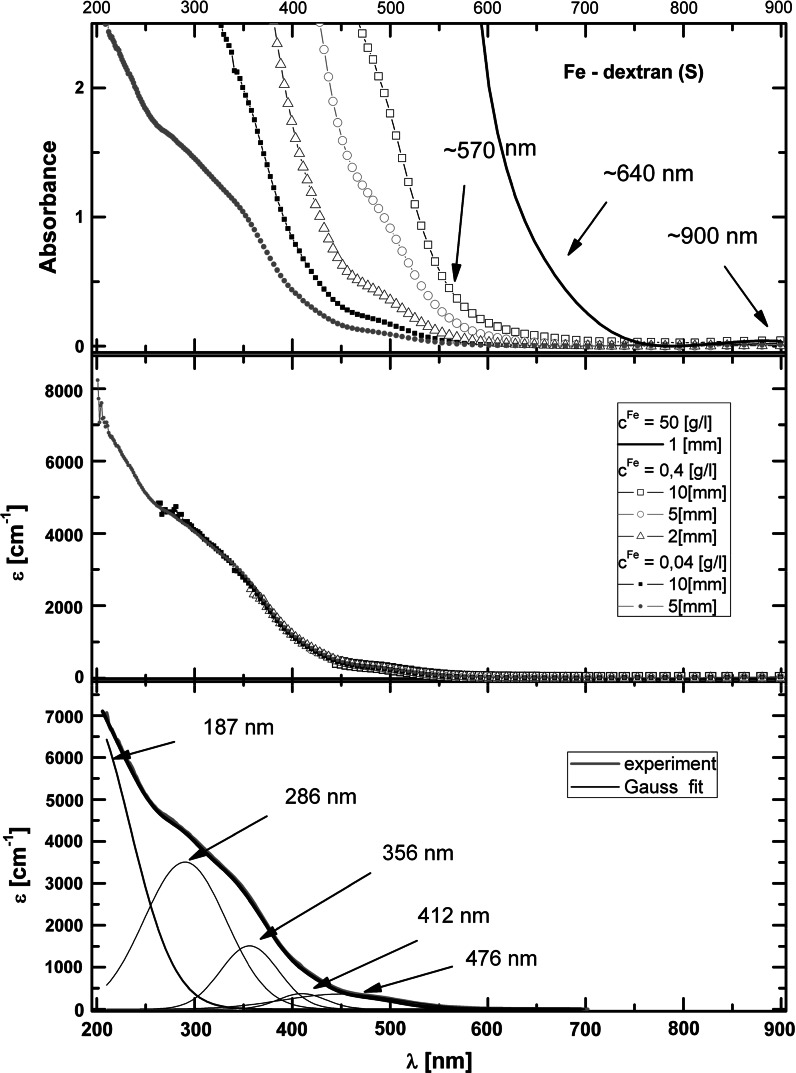
Absorption spectra of Fe-dextran (S) at room temperature. *Top*, absorbance for different iron concentration and cell pathlength the values in nanometers indicate the positions of very weak bands. *Middle*, molar extinction coefficient expressed on the molar iron basis. *Bottom*, fitting of the molar extinction coefficient spectra (*dashed line*) to a Gaussian (*solid line*), with the best-fit Gaussian components indicated. The *arrows* indicate the position of maximum of individual bands obtained from the fit (the size of the *error bar* is smaller than the dimension of the experimental point)

The electronic absorption spectra of the iron oxyhydroxide aqueous suspensions were studied in the spectral range from 200 to 900 nm in the temperature range from 280 to 362 K. All the formulations exhibit a broad featureless absorption extending to the UV. The positions of the relevant bands are indicated by arrows in Fig. 4. The spectral dependence, similar in all the studied compounds, is analyzed in detail for Fe-dextran (S) in Fig. 4.

The data obtained at RT for different iron concentration (from 50 to 0.04 g Fe/L) and pathlength collapse very well into a single curve of the molar extinction coefficient, indicating no anomaly in the concentration dependence (see Fig. 4). Thus, at RT macroscopic aggregation can be judged not to occur and the complexes to be stable in the examined concentration range.

As an aid to the interpretation of the measured absorption we used a series of Gaussians fitted to the spectrum with the error function minimized with respect to the Gaussian height, width, and position. The results are shown in Fig. 4. In general, three groups of bands can by distinguished. The first group is composed of high-intensity bands below 200 nm related to transitions from optical chromophores of the polysaccharide shell (Matsuo and Gekko 2004). Only a part of one band can be seen here, with a maximum at about 187 nm. The second group corresponds to oxo-metal charge transfer transitions of a relatively high-intensity at 286 and 356 nm. The third group includes low-intensity bands above 350 nm arising from a *d*–*d* transition of iron. Bands with a molar extinction coefficient of about 400 M^−1 ^cm^−1^ are seen to occur at 412 and 476 nm, as well as three other very weak transitions (not used in the fitting procedure) at ca. 570, 630, and 900 nm. The latter three only appear as a weak shoulder in solutions of very high concentration (see Fig. 4), with extinction coefficients as low as 10 M^−1 ^cm^−1^. The positions of the bands observed below 500 nm are in a good correlation with the reported spectra of other iron polysaccharide compounds, including iron-gluconate (Kudasheva et al. 2004) and iron-carrageenan (Jones et al. 2000). Also, very well-developed bands were found to occur at 650 and 900 nm for a 27-μm thick crystal of goethite (http://minerals.gps.caltech.edu 2012).

The information acquired from the analysis of the absorption spectra allows to draw conclusions regarding the nature of the iron oxide core of the studied compounds. For low-spin Fe(III) the intensity of the *d*–*d* transition bands should be higher than observed in our study. The presence of Fe(II) would result in a bands in the range from 420 to 450 nm and a high-intensity band at 750 and 950 nm (Fontana et al. 2007), which is not the case here, either (see Fig. 4). The observed low-intensity bands should be associated with characteristic Fe(III) bands in a high-spin state octahedrally coordinated to oxygen (Marusak et al. 1980; Fontana et al. 2007), which are compatible with the expected ferric oxyhydroxide mineral form.

**Fig. 5:**
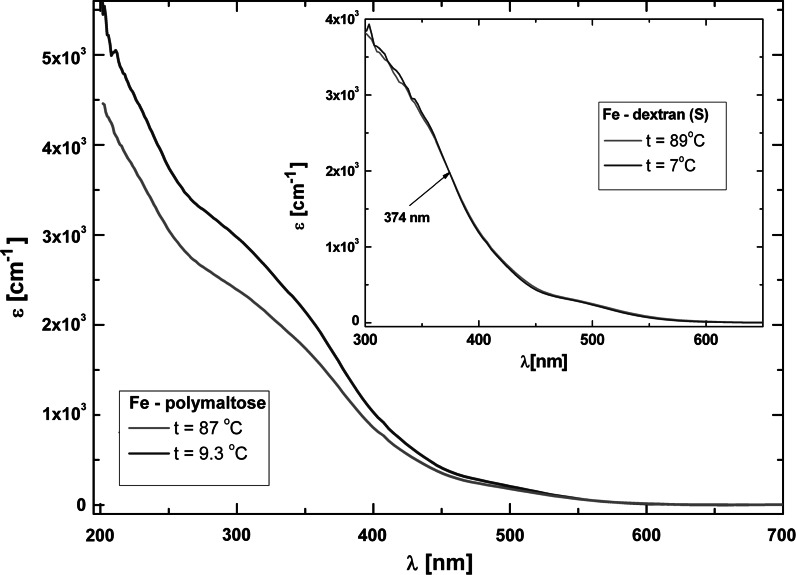
Temperature dependence of absorption spectra of Fe-polymaltose. *Inset* temperature dependence of absorption spectra of Fe-dextran (S); the *arrow* indicates the position of the isosbestic point

A variable temperature UV–Vis optical absorption experiment was performed to determine the effect of the polysaccharide binding on the surface of akaganeite nanoparticles. To illustrate the change in the behavior as a result of heating, in Fig. 5 we present the temperature dependence of the absorption spectra of two of the studied samples, Fe-dextran (S) and Fe-polymaltose. When heated to ca. 363 K, Fe-polymaltose shows an absorption intensity reduced (by ca. 15 % in the 350 nm band, see Fig. 5) with respect to the spectrum taken at 282.3 K. The decrease in the absorption intensity with the temperature is more or less exponential, and the largest changes are observed in the bands below 400 nm, ascribed to the oxo-metal charge transfer. This suggests a degradation of bonds between the core surface and the coating shell, which may affect the stability and the homogeneity of the suspension and lead to particle agglomeration. The core may be dismantled of the shell due to the low polysaccharide concentration in Fe-polymaltose in comparison to the other studied compounds. Therefore, this compound should not be stored much above room temperature. For Fe-dextran (S) and the other two compounds the changes in the absorption spectra upon heating to ca. 363 K are minor (below 2 % in the 350 nm band, see Fig. 5). Any temperature-induced agglomeration is unlikely in these three compounds, which, therefore, are good models for further temperature magnetooptical studies.

#### Magnetically-induced optical birefringence

**Fig. 6:**
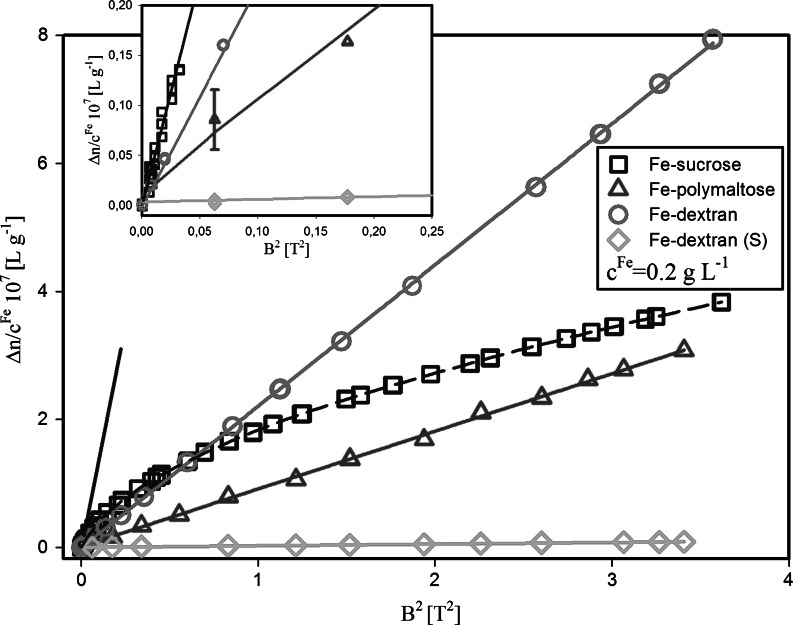
Specific magnetic birefringence Δ*n/c* ^Fe^ versus the *square* of applied magnetic field for iron oxyhydroxide polysaccharide aqueous suspension (iron concentration *c* ^Fe^ = 0.2 g/L). The *solid line* indicates the low-field region; the *dashed lines* serve as eye guides. The *vertical bar* marks the error for experimental points (for clarity the *error bar* is marked for one point only)

**Fig. 7:**
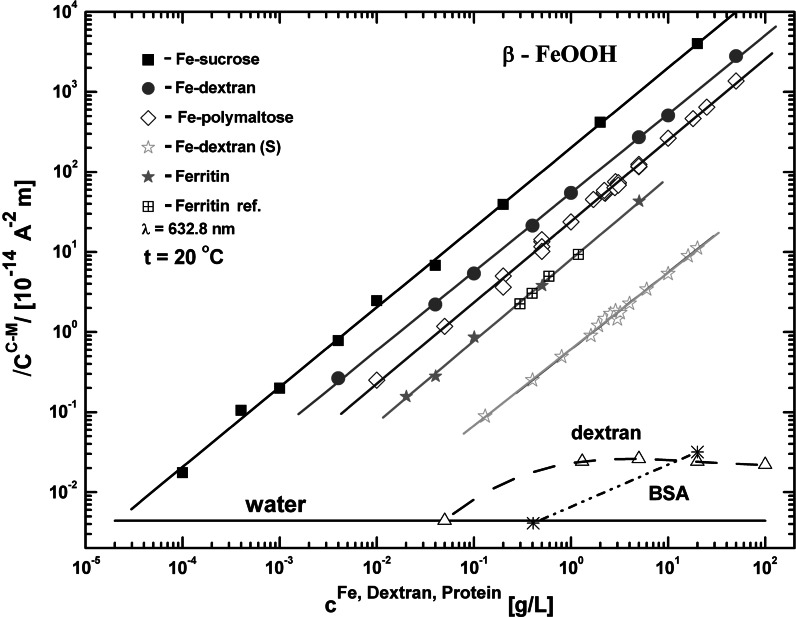
Absolute value of Cotton–Mouton constant *C* ^CM^ versus iron concentration for ferritin and iron polysaccharide aqueous suspensions (Ferritin ref.—data from Pankowska and Dobek 2009). The values of *C* ^CM^ for dextran and bovine serum albumin (BSA) as well as for pure water are indicated, too. The size of the *error bar* is close to the dimension of the experimental point

In all the aqueous suspensions of akaganeite nanoparticles we have studied the magnetically-induced optical birefringence Δ*n* versus the applied magnetic field of up to 2 T (20 kOe). Figure 6 shows a typical behavior of the measured Δ*n* for the iron concentration *c* ^Fe^ = 0.2 g/L. With the help of Eq. (4) and using the low magnetic field birefringence data (see the solid line in Fig. 6), we obtained the value of the Cotton–Mouton constant *C* ^CM^ for each sample. Figure 7 shows concentration plots compared with the characteristics measured for commercial HSF. The specific C–M constant values (i.e., *C* ^CM^/*c* ^Fe^) are listed in Table 2. The effect of the polysaccharide shell is seen to be negligible as the Cotton–Mouton constant measured in aqueous solutions of dextran is very small and only slightly depends on the concentration (*C* ^CM^ ≈ −(0.024 ± 0.004) × 10^−14^ mA^−2^). The value of $C_{\text{Ak}}^{CM}$ obtained for the examined akaganeite nanoparticle suspensions is relatively large (see Table 2), when compared with the values of the Cotton–Mouton constant measured in organic compounds (e.g., nitrobenzene, $C_{\text{Nb}}^{CM}$= 3.32 × 10^−14 ^mA^−2^ (Battaglia and Ritchie 1977)) as well as that for horse spleen ferritin (see Table 2). It is, however, much lower than for aqueous suspension of nanoparticles of other iron minerals. For quite large goethite particles (150 × 25 × 10 nm^3^ nanorods) (Lemaire et al. 2004) the estimated value of $C_{\text{Gt}}^{CM}$ is about 6.11 × 10^−10^ mA^−2^ (for the same amount of iron, i.e., 1 g/L), whereas the magnetic moment per particle was found to be of ca. 1,100 *μ*_B_. The values of the Cotton–Mouton constant measured in magnetite (or maghemite) dispersions (Koralewski et al. 2011b, 2012b) were about five orders of magnitude higher than the $C_{\text{Ak}}^{CM}$values obtained in the present study. This recalls that in the studied akaganeite nanoparticles the contribution to the magnetic birefringence from the permanent magnetic moment should be relatively small.

**Table 2:** Specific Cotton–Mouton constant, optical polarizability anisotropy, magnetic susceptibility anisotropy, magnetic moment, loading factor, and volume density of akaganeite nanoparticles and HSF, with error in parentheses

Property	Fe-sucrose	Fe-polymaltose	Fe-dextran	Fe-dextran (S)	Ferritin
*C*_Sp_^CM^ (10^−14 ^mA^−2 ^Lg^−1^)	203 (3)	24.4 (0.5)	55.1 (1.4)	0.62 (0.02)	8.1 (0.4)^e^
*Δ*α (10^−40 ^Cm^2 ^V^−1^)^a^	10 (3)	80.1	45.3	282	5^f^
Δ*χ* (10^−20 ^JT^−2^)	−6.1/6.4 (0.6)^d^	–	−4.6/4.8^d^	−4.07 × 10^−2^/4.24 × 10^−2d^	3.8^f^
*μ*_m_ (*μ*_B_)	2,157/239^d^	–	1,636/181^d^	164/18^d^	(128–556)^g^
LF^b^	337	2,700	1,560	9,540	1,600
*ρ* ^N^(10^21 ^m^−3^)^c^	30.4	3.8	6.6	1.07	~6

^a^The product Δ*α*Δ*χ* for iron sucrose is negative; the sign of Δ*α* was chosen arbitrarily

^b^Calculated for core diameter as in Table 1 (TEM)

^c^For *c* ^Fe^ = 1 g/L

^d^For the second method of linearization, see text

^e^Specific Cotton–Mouton constant for apoferritin and ferritin with LF = 2200 is equal to *C*_sp_^CM^ = (0.018 ± 0.004) × 10^−14 ^mA^−2^ and *C*_sp_^CM^ = (6.97 ± 0.30) × 10^−14 ^mA^−2^, respectively

^f^Data estimated from (Pankowska and Dobek 2009)

^g^Estimated from low-temperature magnetization measurements (Brem et al. 2006)

The dependence of *C* ^CM^ on the iron concentration is clearly linear in a very wide concentration range (more than five decades, see Fig. 7). This shows that the nanoparticles only interact magnetically with the external field. No inter-particle interaction occurs even at the highest concentration examined, which suggests a relatively small magnetic moment of the particles.

The results presented in Fig. 7 also indicate that the presence of ferritin (or its mimetics) in an unknown solution can be confirmed down to the relatively low iron concentrations of the order of 10^−3^–10^−4^ g/L. The measurement sensitivity can be increased still further by two or three orders of magnitude by using a more precise polarimeter (see for example dual channel balanced photodetector system described by Ku et al. (2008) and performing the measurements in higher magnetic field, which nowadays may reach 11 T using commercial superconducting split magnets (for example see Oxford Instruments Spectromag system (http://www.oxford-instruments.com 2013)). This may open a possibility for detecting and tracking non-heme metaloproteins in serum or human body fluids. Thus, the linear relation between the C–M constant and the iron concentration in a wide range, together with discrimination of mineral core provides a basis for possible analytical applications of the C–M effect in biomedicine. It is important to note that recently rapid detection method for malaria diagnosis based on magnetooptical phenomena discussed here was presented and tested (Newman et al. 2008; Mens et al. 2010). Quite recently some further improvement in detection of malaria pigments based on magnetically-induced birefringence/dichroism measurements have been proposed lately (Butykai et al. 2012).

Only in the case of Fe-sucrose the magnetic birefringence is seen to depart from the simple quadratic dependence, which indicates the beginning of the saturation process (Fig. 6). Therefore, solely for this sample we used a graphical method of estimation of the value of Δ*n*_s_, described in "Theoretical background" section. The approximate Δ*n*_s_ value (normalized to 1 g/L iron concentration) obtained for Fe-sucrose in this approach is about 7 × 10^−7^. Assuming the akaganeite density (Cornell and Schwertmann 2003) and the particle size as specified in Table 1, we obtained the volume fraction and the volume concentration *ρ* ^N^. Using these values and Eq. (2) we calculated the optical polarizability anisotropy per particle to obtain approximate value Δ*α* = 10 × 10^−40^ Cm^2 ^V^−1^. On the assumption that this value is a material constant for akaganeite, the values for other iron oxyhydroxide polysaccharide suspensions were estimated from the relation (Δ*α*)^Fe-polysaccharide^ = (LF)^Fe-polysaccharide^ × (Δ*α*)^Fe-sucrose^/337, where LF is the number of Fe atoms per grain and 337 is LF for Fe-sucrose. The results are presented in Table 2.

**Fig. 8:**
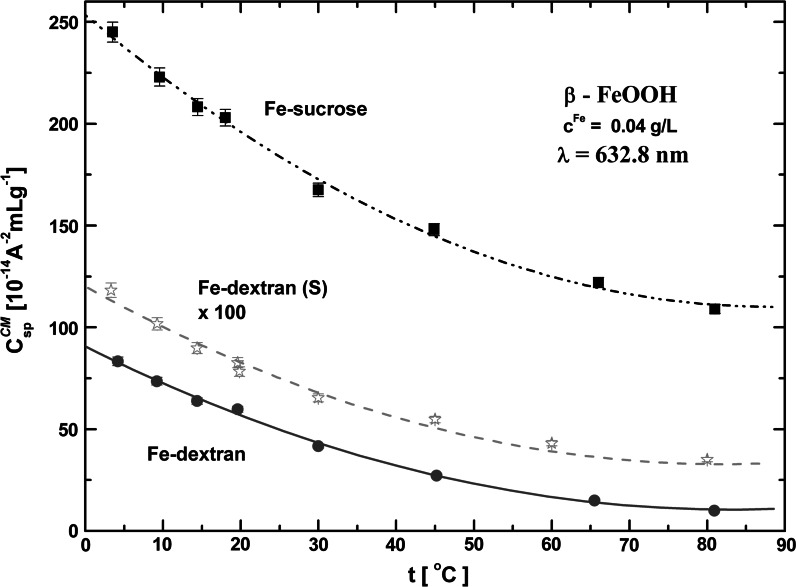
Temperature dependence of the specific Cotton–Mouton constant for iron oxyhydroxide polysaccharide aqueous suspensions with iron concentration *c* ^Fe^ = 0.04 g/L. The values of *C* ^CM^ for Fe-dextran (S) have been magnified by the factor of 100. The *vertical bar* marks the error for experimental points

**Fig. 9:**
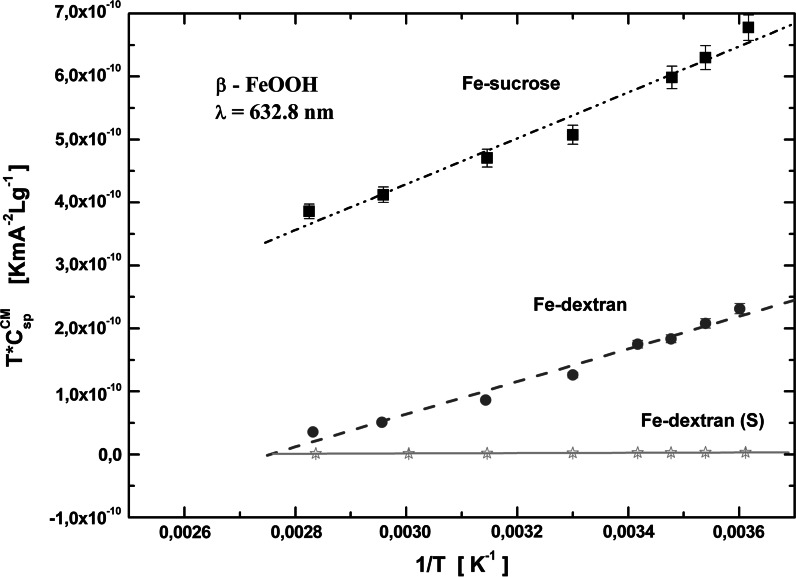
Product *TC* ^CM^ versus 1/*T* for iron oxyhydroxide polysaccharide aqueous suspensions; the *lines* are the best fit by the linear equation. The *vertical bar* marks the error for experimental points

**Fig. 10:**
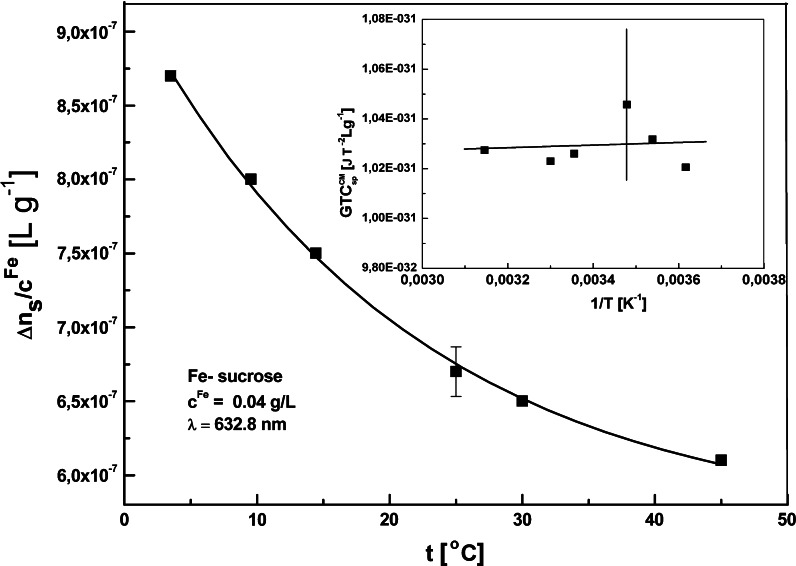
Temperature dependence of the specific saturation magnetic birefringence Δ*n*_s_ for the Fe-sucrose aqueous suspension (*c* ^Fe^ = 0.04 g/L, *λ* = 632.8 nm). In the *inset*, the product *GTC* ^CM^ versus 1/*T*; the *line* is the best fit by the *linear* equation. The *vertical bar* marks the error for experimental points (for clarity the *error bar* is marked for one point only)

The induced magnetic birefringence was measured in different temperature conditions, and *C* ^CM^ was calculated for each temperature. However, the Fe-polymaltose sample was excluded from these measurements because of its low thermal stability. The results for the specific *C*_sp_^CM^ (i.e., *C* ^CM^/*c* ^Fe^) obtained for the other three PIC suspensions are shown in Fig. 8. The temperature dependence of the Cotton–Mouton constant is clearly nonlinear. The results were linearized (by the first method) by plotting the product *TC* ^CM^ versus 1/*T* (see Fig. 9), which allowed the determination of Δ*χ* and *μ*_m_ of the particle from the intercept and the slope of the linear dependence by using the estimate values of Δ*α* (see Table 2). In the case of Fe-sucrose we were also able to estimate the saturation birefringence (Δ*n*_s_) for each measurement temperature (Fig. 10), and found that the temperature dependence of Δ*α* was not negligible. In that case the second method of linearization yielded different values of Δ*χ* and *μ*_m_ (see Table 2), which describe well the magnetic properties of Fe-sucrose. It needs to be stressed that both linearization procedures are subject to relatively large error due to data collection, and can only be used in a relatively narrow temperature range far from 0 K. The observation of full saturation birefringence in the other PIC samples would require very high magnetic fields (much higher than those used in this experiment). The estimate (see below) value of saturation magnetic field *B*_S_ is ca. 30 T. Note that the highest magnetic field in which measurements of the C–M constant have been performed to date had a magnitude of ca. 20 T (Gielen et al. 2009). We hope that studies in stronger fields will be possible in the near future. At the moment, we can assume that for the other PIC compounds the temperature dependence of Δ*n*_s_ is similar as for Fe-sucrose. The values of Δ*χ* and *μ*_m_ extracted on this assumption are specified in Table 2. The Δ*χ* values for akaganeite nanoparticles are of the same order of magnitude as those for HSF (see Table 2), but much higher than those for single organic molecules with a high anisotropy of magnetic susceptibility, such as benzene (Δ*χ* ~ −1 × 10^−27^ JT^−2^) or porphyrin ring (Δ*χ* ~ −1 × 10^−26^ JT^−2^) (Maret and Dransfeld 1985).

The signs of saturation shown by the Fe-sucrose sample provided a basis for a tentative interpretation of the results in the Langevin formalism. The best fit to the experimental data by the Eq. (6a) (for a discrete particle diameter, i.e., monodisperse nano-ferrofluids) is obtained for the average magnetic moment of the particle *μ*_m_ ≈ 1,191 μ_B_ (see Table 4, second column). This value is several times higher than that calculated by the second method of linearization; also the average diameter of the core is much higher in comparison to the XRD and TEM data. The quality of the fit is not very high, either. The TEM images (Fig. 2) indicate this could be related to the particle size distribution. Since the estimation of size of roughly spherical particles used in the magnetic measurements relies on Eq. (7), the saturation magnetization *M*_s_ of the material particle should be known. We attempted to calculate *M*_s_ from the available experimental magnetization data (Gutierrez et al. 2005) for Fe-sucrose.

**Fig. 11:**
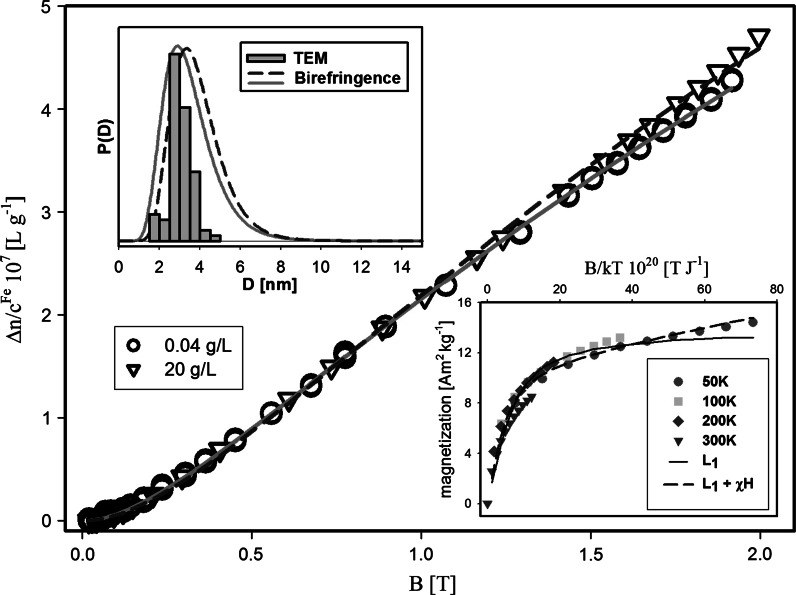
Specific magnetic birefringence Δ*n*/*c* ^Fe^ as a function of the applied magnetic field for Fe-sucrose aqueous suspension (*c* ^Fe^ = 0.04 and 20 g/L). The *dashed* and *solid lines* are the best fit by the Langevin function by Eqs. (6) and (9).* Top inset* particle size distribution obtained in TEM and magnetic birefringence measurements. *Bottom inset* magnetization versus *B*/*kT*; the *dashed* and *solid lines* are the best fit to the experimental points (data from Gutierrez et al. 2005) by the usual Langevin function and the usual Langevin function plus the *linear* term *χH*, respectively. The size of the *error* *bar* is close to the dimension of the experimental point

The inset in Fig. 11 shows the magnetization *M* plotted versus *B*/*kT*. The data for different values of *T* collapse well into a single curve, suggestive of a temperature-independent *μ*_m_. The experimental points have been fitted by the usual Langevin function *L*_1_(*p*) with *μ*_m_ and *M*_s_ used as fitting parameters (see the inset in Fig. 11). The agreement is seen to be slightly better when the experimental magnetization *M* is described by the sum of the Langevin function and a linear contribution to the magnetization, χ*H*. Therefore, we have fitted also the expression: *M* = *M*_s_ *L*_1_(*p*) + χ*H*. The fitting parameters are collected in Table 3. The value of* χ* obtained for Fe-sucrose akaganeite nanoparticles is of the same order as that reported for HSF (Zborowski et al. 1995; Makhlouf et al. 1997). The obtained value of *M*_s_, ranging from 11 to 13.9 Am^2 ^kg^−1^, seems rather high for the antiferromagnetic akaganeite. However, there are no other literature data for this particular material for comparison. We can compare this value with *M*_s_ = 2.5 Am^2 ^kg^−1^ reported for 7 nm nanoparticles of a PIC compound marketed under the name Niferex (Mohie-Eldin et al. 1994). Slightly smaller (5 nm) ferrihydrite particles, pure or doped with Ni, Mo, and Ir, were found to have *M*_s_ in the range from 6 to 9 Am^2 ^kg^−1^ (Punnoose et al. 2004). In this context, the relatively high value of *M*_s_ found for Fe-sucrose can be correlated with the very small diameter (~3 nm) of the mineral core.

**Table 3:** The parameter values obtained in the fitting procedure with different types of function describing the magnetization for Fe-sucrose; *χ* ^2^ describes the goodness of the fit

Type of function	*L*_1_(*p*)	*L*_1_(*p*) + *χH*
*M*_s_ (Am^2 ^kg^−1^)	13.9	11.41
*D* (nm)	4.67	5.70
*μ*_m_ (μ_B_)	319	467
*χ* (10^−7 ^m^3 ^kg^−1^)	na	0.151
*χ* ^2^	0.625	0.411

*na* not applicable

It is important to note here that the measured values of magnetization of Fe-dextran (S) (Gutierrez and Lazaro 2007) are more than 10 times lower than those of Fe-sucrose. At the same time the very weak departure from linearity of the experimental *M*(*B*) dependence (Gutierrez and Lazaro 2007) clearly shows that saturation should be expected in very high magnetic field. The situation is similar to that observed for ferritin where full saturation of magnetization has not been observed even in the field as high as 55 T (Guertin et al. 2007; Silva et al. 2009). Thus, the average value of *μ*_m_ for Fe-dextran (S) can be expected to be much smaller than that for Fe-sucrose. This relation is in very good agreement with the estimated values of *μ*_m_ specified in Table 2.

The experimental values of magnetic birefringence of Fe-sucrose were fitted by the Langevin function described by the Eq. (6) and (6a) with a log-normal distribution (see Eq. (9)) and the assumed value of *M*_s_ = 13.9 Am^2 ^kg^−1^. The effect of the Δ*n*_s_ value on the goodness of fit with a specific Langevin function was tested, too. The respective values of fitting parameters are collected in Table 4. The fits are seen to be better for Δ*n*_s_ much higher (~20 × 10^−7^) than the value obtained by the graphical approximation method (~7 × 10^−7^). The obtained values of 〈*D*〉 and their distribution, shown in Fig. 11, are in good agreement with the diameter obtained from XRD and TEM (see Table 4, columns 4 and 5, and Fig. 11). Also 〈*μ*_m_〉 obtained for high Δ*n*_s_ in the fitting procedure is in good agreement with the value resulting from the second method of linearization of the temperature dependence of the C–M constant (see Tables 2, 4).

**Table 4:** The parameter values obtained in the fitting procedure with different types of Langevin function for Fe-sucrose (*M*_s_ = 13.9 Am^2 ^kg^−1^), with average magnetic moment 〈*μ*_m_〉, average diameter 〈*D*〉, its standard deviation *σ* and the coefficient *R* ^2^ or *χ* ^2^ describing the goodness of the fit

Type of function	*L*_2_(*p*,*q*)	*L*_2_(*p*,*q*)	*L*_2_(*p*)	*L*_2_(*p*)	*L*_2_(*p*,*q*)
Δ*n*_s_ (10^−7^)^a^	7	7.34	7	20	20
*D*_0_ (nm)	6.2	na	7.52	3.28	2.95
*s*	0.24	na	0.0012	0.35	0.69
〈*D*〉 (nm)	6.4	7.24	7.52	3.49	3.74
*σ* (nm)	1.56	na	0.1	1.28	2.92
〈*μ*_m_〉 (μ_B_)	823	1,191	1,135	133	159
*Δχ* (10^−21^ JT^−2^)	6.21	0.62	na	na	4.56
*R* ^2^/*χ* ^2^	0.9988	0.9981	1.262	0.103	0.9995

*na* not applicable

^a^At RT and *λ* = 632.8 nm, for *c* ^Fe^ = 1 g/L

In the estimation based on the best-fit parameters (see Table 4, column 4) the magnitude of the magnetic field corresponding to 80 % of birefringence saturation is found to be of ca. 30 T. This means that any future validation of the values of magnetic characteristics of Fe-sucrose obtained in isothermal experiment will require fields of magnitude as high as 20–30 T. This is not a simple task, though. Alternatively, we have shown that good information on the anisotropy of susceptibility and the magnetic moment of the studied compounds can be obtained from temperature measurements with a field of the order of only a few Tesla’s.

In the mineral core of Fe-sucrose the estimated magnetic moment per particle was found to be about 133–239 *μ*_B_, depending on the analysis method used. These values are similar to those reported in the literature for HSF (Kilcoyne and Cywinski 1995; Brem et al. 2006) (see Table 2). Although bulk akaganeite, being an antiferromagnet below room temperature, should not possess bulk magnetization, also magnetic properties are known to strongly depend on the particle size. In fact, HSF and the studied akaganeite suspension show superparamagnetic properties, as observed by the VSM method. (Neel 1961) showed that very small antiferromagnetic particles do exhibit a net magnetic moment resulting from incomplete surface spin compensation. The effect occurs when the surface spins outnumber the volume ones. This *surface*-*to*-*volume* effect is seen well in the compound studied: the estimated *μ*_m_ decreases with increasing size of the nanoparticles (see Tables 1, 2). For fine nanoparticles with a diameter below ~5 nm the number *N*_u_ of uncompensated spins is of the order of (*N*_p_)^1/2^, where *N*_p_ is the number of magnetic ion spins per particle (Neel 1961). Assuming the theoretical value of magnetic moment of Fe^3+^ equal to 5.92 *μ*_B_, from this relation we get the value of magnetic moment per particle of ca. 110 *μ*_B_ for Fe-sucrose, which is in quite good agreement with the experimentally obtained value of 133 *μ*_B_.

**Fig. 12:**
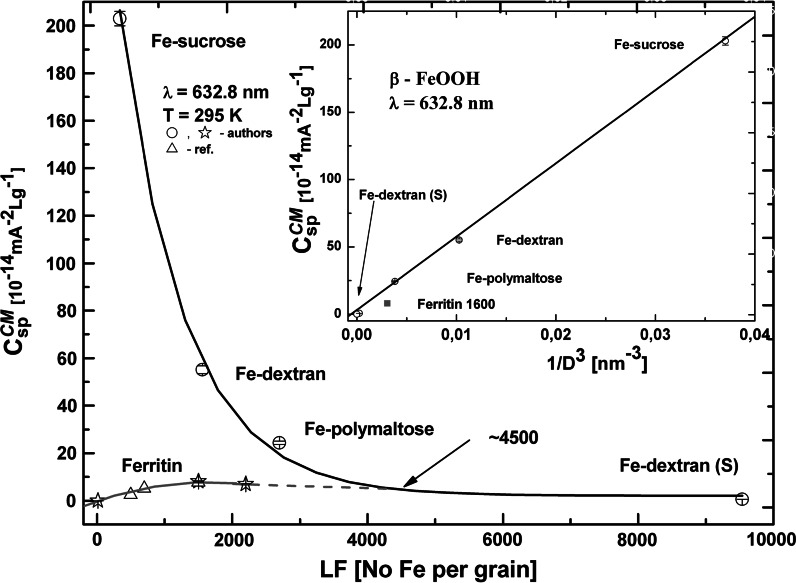
Specific Cotton–Mouton constant versus the number of Fe atoms per grain of iron oxyhydroxide polysaccharides and LF of ferritin (ref. data from Pankowska and Dobek for reconstitution of HSF core). *Inset* *C* ^CM^ versus the inverse cube of the nanoparticle diameter (*D* = 7 nm for HSF). The *vertical bar* marks the error for experimental points

Using Eq. (5) and the values of Δ*χ* and *μ*_m_ we can compare their relative contributions to the measured value of *C*_sp_^CM^. For Fe-sucrose the contribution Δ*C*_Δχ_ from the magnetic susceptibility anisotropy is ca. 60 times higher than the contribution Δ*C*_μm_ from the permanent magnetic moment of the nanoparticle, i.e., Δ*C*_Δχ_ ≫ Δ*C*_μm_. With the actual nanoparticle size used in the Eq. (5) (particle volume fraction *ϕ* = *ρ* ^N^ *V* and *μ*_m_ = *M*_s_ *V*) we can distinguish two general relations describing the C–M constant for spheroid particles with low eccentricity, in which case the particle volume can be approximated by *V* = π*D* ^3^/6. One relation is *C* ^CM^ ∝ 1/*D* ^3^, and applies to Δ*C*_Δχ_ ≫ Δ*C*_μm_. The other one is *C* ^CM^ ∝ *D* ^3^, and corresponds to the opposite situation, i.e., Δ*C*_μm_ ≫ Δ*C*_Δχ_. Of course, in the case of nanoparticles in the form of a disk or a rod the relation between *C* ^CM^ and the main dimensions of the particle will be different. The studied nanoparticle suspensions are found to fulfill the relation *C* ^CM^ ∝ 1/*D* ^3^ (see Fig. 12), which is consistent with the antiferromagnetic properties of HSF and akaganeite nanoparticles (Cornell and Schwertmann 2003).

Figure 12 shows the dependence of the specific Cotton–Mouton constant on the number of Fe atoms per grain (load factor LF in the case of ferritin), in the studied akaganeite nanoparticle aqueous suspensions, against the similar results obtained after reconstruction of the mineral core of HSF. Ferritin is seen not to follow the dependence observed for massive grains of akaganeite nanoparticles. In HSF the value of *C*_sp_^CM^ grows with increasing LF and for ca. 1,600–2,000 Fe atoms per grain reaches a value corresponding to that for ca. 4,500 Fe atoms per grain in massive akaganeite particles. The number of 4,500 Fe atoms is known to correspond to fully loaded ferritin. This means that ferritin only needs ca. 1,600 Fe ions to show the magnetic behavior characteristic of a massive nanoparticle of the protein cavity volume. This may be possible if the iron atoms form a hollow sphere inside of the apoferritin cavity. Taking into account the ferrihydrite crystallographic data, one may easily calculate that the number of Fe atoms necessary for a 1-nm thick ideal hollow sphere with an external diameter of 7 nm is about 1,500–1,600. This means that the ferritin core behaves like a non-Euclidian solid in comparison to the grain formed by akaganeite particles. This observation may corroborate the diffusion-limited aggregation (DLA) model of the ferritin core structure proposed by some authors (Frankel et al. 1991; Harris et al. 1999). It also seems to be in accordance with a more precise model of ferritin core very recently proposed by several authors (Pan et al. 2009; Ciasca et al. 2011). The electron microscopy study carried out by Pan et al. confirms that the mineral core of human hepatic ferritin is composed of surface-disordered ferrihydrite subunits connected to a lower-density center. When the core is not fully loaded a reduction of density, or even a hole, in the center of the mineralized core can be expected to occur (Pan et al. 2009). A similar observation has been made on the basis of very recent small angle X-ray scattering (SAXS) measurements during iron release, used for modeling the cross-sectional shape of ferritin as a function of the metal content (Ciasca et al. 2011). Ciasca et al. suggest that the mineralization process starts from the shell and proceeds toward the center of the cavity. As the metal content increases the hole radius diminishes to vanish completely in the end. Also worthy of notice is the recent experimental observation of a hollow geometrical structure of the artificial Co_3_O_4_ ferritin (Kim et al. 2005).

## Conclusions

The present paper is the first to report measurements of magnetic birefringence and its temperature dependence for akaganeite nanoparticles coated by a polysaccharide aqueous suspension (PIC). The measurement results are compared with the respective data we have obtained for horse spleen ferritin. We have also discussed the structural and magnetic properties studied by XRD, TEM, and VSM. The temperature dependence of the refractive properties as well as the UV–Vis absorption spectra provide further information on the stability of the formulation and the composition of the mineral core. The values of optical polarizability anisotropy and magnetic susceptibility anisotropy have been obtained from the temperature dependence of the saturation magnetic birefringence and the Cotton–Mouton constant, respectively. In the Fe-sucrose sample, the suspension with the smallest akaganeite nanoparticles, the isothermal dependence of the magnetic birefringence on the magnetic field has been described tentatively by different types of Langevin function to provide another way of estimation of the Δ*χ* and *μ*_m_ values. The net magnetic moment per particle has been found to be relatively small and to increase with decreasing grain diameter. This *surface*-*to*-*volume* effect observed for the studied compounds is consistent with the superparamagnetic behavior of the antiferromagnetic nanoparticles. The established relation between the C–M constant and the diameter of the nanoparticles confirms the dominant contribution of the magnetic susceptibility anisotropy Δ*χ* to the net magnetic birefringence of the studied compounds. The PICs and HSF show consistency in the magnetic behavior, which means that the studied hematinic formulations can be used as mimetic models of ferritin. The results provide evidence that the ferritin core has a structure of a non-Euclidian solid, which corroborates latest literature reports. Although the elucidation of the mineralization and demineralization processes requires further studies, these findings may be of help in the investigation of the possible role of ferritin in some diseases. The presented results show that magnetic birefringence/Cotton–Mouton effect measurements can provide an important tool for recognizing the magnetic properties of biologically relevant substances.
